# The RNA binding protein RBMS3 inhibits the metastasis of breast cancer by regulating Twist1 expression

**DOI:** 10.1186/s13046-019-1111-5

**Published:** 2019-02-28

**Authors:** Lei Zhu, Pei-Wen Xi, Xiao-Xia Li, Xi Sun, Wen-Bin Zhou, Tian-Song Xia, Liang Shi, Yue Hu, Qiang Ding, Ji-Fu Wei

**Affiliations:** 1 Jiangsu Breast Disease Center, the First Affiliated Hospital with Nanjing Medical University, 300 Guangzhou Road, Nanjing, 210029 China; 2 Research Division of Clinical Pharmacology, the First Affiliated Hospital with Nanjing Medical University, 300 Guangzhou Road, Nanjing, 210029 China; 3 Department of Critical Care Medicine, The First Affiliated Hospital with Nanjing Medical University, 300 Guangzhou Road, Nanjing, 210029 China

**Keywords:** Breast cancer, RBMS3, Twist1, MMP2, Metastasis, mRNA stability

## Abstract

**Background:**

Metastasis remains the biggest obstacle for breast cancer treatment. Therefore, identification of specific biomarker of metastasis is very necessary. The RNA binding protein 3 (RBMS3) acts as a tumor suppressor in various cancers. Whereas, its role and underlying molecular mechanism in breast cancer is far from elucidated.

**Methods:**

Quantitative real-time PCR and western blots were carried out to determine the expression of RBMS3 in breast cancer cells and tissues. Transwell and in vivo metastasis assay were conducted to investigate the effects of RBMS3 on migration, invasion and metastasis of breast cancer cells. Transcriptome sequencing was applied to screen out the differential gene expression affected by RBMS3. RNA immunoprecipitation assay combined with luciferase reporter assay were performed to explore the direct correlation between RBMS3 and Twist1 mRNA.

**Results:**

RBMS3 was downregulated in breast cancer and ectopic expression of RBMS3 contributed to inhibition of cell migration, invasion in vitro and lung metastasis in vivo. Furthermore, RBMS3 negatively regulated Twsit1 expression via directly binding to 3′-UTR of Twist1 mRNA, and thereby decreased Twist1-induced expression of matrix metalloproteinase 2 (MMP2). Additionally, Twist1-induced cell migration, invasion and lung metastasis could be reversed by the upregulation of RBMS3.

**Conclusions:**

In summary, our study revealed a novel mechanism of the RBMS3/Twsit1/MMP2 axis in the regulation of invasion and metastasis of breast cancer, which may become a potential molecular marker for breast cancer treatment.

## Background

Breast cancer is the most frequently diagnosed malignant tumor among women in the world [1]. Despite the great progress has been made in the prevention, detection and adjuvant treatment of breast cancer in recent years, metastasis is responsible for the low 5-year overall survival rate of metastatic breast cancer patients and remains the biggest obstacle for breast cancer treatment [2]. In order to design more effectively strategy to prevent breast cancer metastasis, the deep exploration on the molecular mechanism and identification of specific biomarker of this process is very necessary.

As we know, metastasis is quite a complicated process which account for 90% of cancer death [3]. At present, the multiple steps of tumor metastasis include detachment, invasion, intravasation, extravasation and proliferation [4]. Among these steps, the underlying molecular and biological programs might include epithelial mesenchymal transition (EMT) [5] and tumor microenvironment [6]. EMT could be triggered by a set of transcription factors, such as Zeb1, Zeb2, Twist1, Snail, Slug [5]; and signaling pathways, including Wnt [7], transforming growth factor-β (TGF-β) [8], Interleukin 6 (IL-6) [9], and epidermal growth factor receptor (EGFR) signaling [10]. Subsequently, activation of these transcription factors enhance tumor cell invasion ability to breakdown physical barriers, such as the extracellular matrix (ECM) and the basal membrane, by promoting the secretion of extracellular proteases, including matrix metalloproteinases (MMPs) family [11]. For breast cancer, ectopic secretion of MMPs family, such as MMP-1, MMP-2 and MMP-9, as well as VEGF, angiopoietin-like-4 (Angptl4), and COX-2, could degrade and destroy vascular endothelial cell junctions to promote tumor cells metastasis [12, 13]. Evidences indicated that some RNA binding proteins (RBPs) are the critical regulators involved in this process. For example, the RNA binding motif protein 38 (RBM38), a tumor suppressor in breast cancer, was reported to be involved in the TGF-β signaling pathway and inhibit EMT by rescuing the expression of zonula occludens-1 to prevent cancer metastasis [14]. The RNA binding motif protein 47 (RBM47) inhibited breast cancer metastasis by regulating DKK1 expression via Wnt pathway [15]. RBPs are a sort of proteins which can bind to the 3′-untranslated region (3′-UTR) of mRNAs of target genes and regulate their expression at post-transcriptional level, including: RNA splicing, polyadenylation, modification, stability and translation. Although RBPs play the key role in various biological processes, only a small proportion of them have been well studied [16].

Recently, an RBP, the RNA binding motif single stranded interacting protein 3 (RBMS3) is found to be located at 3p24-p23, where is often found deleted or mutated in cancers, suggesting its potential role in tumor suppressing [17]. Moreover, downregulation of RBMS3 in esophageal squamous cell carcinoma [18], lung squamous cell carcinoma [19], nasopharyngeal carcinoma [20] and gastric carcinoma [21] are frequently correlated with poor prognosis in patients and loss of RBMS3 contributed to chemoresistance in epithelial ovarian cancer [22]. Furthermore, RBMS3 was found to inhibit the proliferation and tumorigenesis of breast cancer cells, at least in part, through inactivation of the Wnt/β-catenin signaling pathway [23]. However, there were few studies on the role of RBMS3 in the metastasis of breast cancer and related mechanism.

In the present study, we demonstrated that RBMS3 played a critical role in the metastasis of breast cancer. To better understand the mechanism in the process, transcriptome sequencing was applied to identify the differential gene expression affected by RBMS3. We provided a novel mechanism that the basic helix-loop-helix transcription factor Twsit1, the key regulator in cancer metastasis, was regulated by RBMS3 in breast cancer cells in vitro and in vivo. Furthermore, as MMP2 is the direct downstream target of Twist1 [24], repression of Twist1 resulted in downregulation of MMP2 expression, thereby inhibiting the metastasis of breast cancer cells.

## Methods

### Cell cultures

MDA-MB-231, MDA-MB-453, SUM-1315, SKBR3 and ZR-75-1 cell lines (ATCC, USA) were cultured in Dulbecco’s modified eagle medium (Wisent, China) supplemented with 4.5 mg/ml glucose, 10% fetal bovine serum (Gibco, USA), 100 μg/ml penicillin, and 100 μg/ml streptomycin (Hyclone, USA). All cells were incubated in a humidified atmosphere containing 5% CO_2_ at 37 °C.

### Clinical tissues

The collection and use of tumor tissues and adjacent normal tissues were reviewed and approved by the ethical committee of the First Affiliated Hospital of Nanjing Medical University. These tissues were stored in liquid nitrogen for mRNA and protein detection (Reviewer #1 comment 4).

### Lentivirus transfection

MDA-MB-231 and SUM-1315 cells were transfected with lentivirus (Genepharm, Shanghai, China) to overexpress RBMS3, Twist1 (full-length cDNA sequence) (Reviewer #1 comment 9), or repress RBMS3 expression. Puromycin (3 μg/ml) was used to select the stable cells for two weeks.

### RNA isolation, reverse transcription and quantitative real-time PCR (qRT-PCR)

TRIZOL reagent (TaKaRa, Kusatsu, Japan) was used for the isolation of total RNA. Reverse transcription and qRT-PCR were carried out as described [25]. The sequences of primers were listed in Table 1 (Reviewer #2 comment 3).

**Table 1:** The primers sequences of related genes used in qRT-PCR

Genes	Forward (5′- to 3′-)	Reverse (5′- to 3′-)
β-actin	TCACCCACACTGTGCCCATCTACG A	CAGCGGAACCGCTCATTGCCAATGG
RBMS3	GCATCTCTCAAGGCAAATGG	CAACACCTCTGCTGACTCCA
MMP2	GTGATGGTGTCTGCTGGAAA	GGAAGCAAACCTCGAACAGA
Twist1	GGGCCGGAGACCTAGATG	TTTCCAAGAAAATCTTTGGCATA

### Western blot analysis

The immunoblots were processed as described previously [26]. The primary antibodies included anti-mouse RBMS3 (Sigma-Aldrich, St Louis, MO, USA), β-actin (Cell Signaling Technology, USA), Twist1 (Abcam, Cambridge, MA, USA), anti-rabbit MMP2 (Abcam). The secondary antibodies were purchased from Cell Signaling Technology. The dilutions of antibodies were according to the product usage information.

### Conditioned media

5 × 10^5^ cells were seeded into a six-well and supplied with DMEM contained 10% FBS. On the other day, the media in each well was replaced by 2 ml of serum and antibiotic-free DMEM. After incubation for 2 days, the media was filtered by 0.22 mm filters and collected for further use. Western blot was performed to analyze the expression of MMP2 in the media.

### Transwell migration and invasion assay

The migration and invasion assay of SUM-1315 and MDA-MB-231 cells were conducted as described previously [14].

### Experimental metastasis assay

The 4-week-old balb/c female nude mice were obtained from Model Animal Research Center of Nanjing University (Nanjing, China) and the animal use was approved by Institutional Animal Care and Use Committee of Nanjing Medical University. For metastasis assay, SUM-1315-luc cells (2 × 10^6^ /0.2 ml) expressing RBMS3, Twist1, or Twist+RBMS3 were injected into the tail veins of the nude mice. Every two weeks, the mice were anesthetized and injected intraperitoneally with 0.2 ml of Nano-Glo luciferase assay (Promega, USA). Five minutes after injection, the IVIS Illumina System (Caliper Life Sciences) was applied for imaging. After 8 weeks, the mice were sacrificed and examined for lung metastases using Hematoxylin-Eosin (H&E) staining.

### Transcriptome analysis

3 × 10^6^ well-conditioned RBMS3-overexpressing and RBMS3-control of SUM-1315 cells were isolated for total RNA. Each group was in triplicate. Then, the transcriptome sequencing was conducted by Beijing Genomics Institute (Wuhan, China) using Illumina HiSeq 4000 Systems. Reads were trimmed (Cutadapt, version 1.1.6) and mapped (TopHat2, version 2.1.1) to the human transcriptome. Then, RPKM (reads per kilobase per million mapped reads) values were estimated by using Cufflinks (version 2.2.1).

### The cancer genome atlas data analysis

The Cancer Genome Atlas (TCGA) data were obtained from the cBio cancer genomics portal (http://www.cbioportal.org/). Then, the expression data were analyzed by R (version 3.4.1) and UCSC Xena (https://xena.ucsc.edu/welcome-to-ucsc-xena/).

### Luciferase reporter assay

Briefly, cells were seeded into 24-well plate and transfected with Renilla luciferase vector and pGL3 reporter. After 48 h, the luciferase activity was measured by the Dual-Luciferase Reporter Assay System (E1910, Promega, Madison, WI, USA). All the experiments were repeated at least three times.

### RNA immunoprecipitation (RIP)

RIP assay was performed as previously described [27]. The protein A/G magnetic beads were used to elute immunocomplexes. After purification, the purified RNA was analyzed by RT-PCR and qRT-PCR.

### Statistical analysis

All experiments were performed in triplicate, whenever applicable. Student’s t-test and one-way analysis of variance were performed by Graphpad Prism 7.0 Software (GraphPad, La Jolla, CA, USA) to analyze the data sets, which were continuous variables. The survival curve was generated by Kmplotter (www.kmplot.com). All data were presented as mean ± standard error of the mean (SEM). *P* < 0.05 was considered statistically significant.

## Results

### RBMS3 expression was downregulated in human breast tumors and correlated with poorer prognosis

**Fig. 1:**
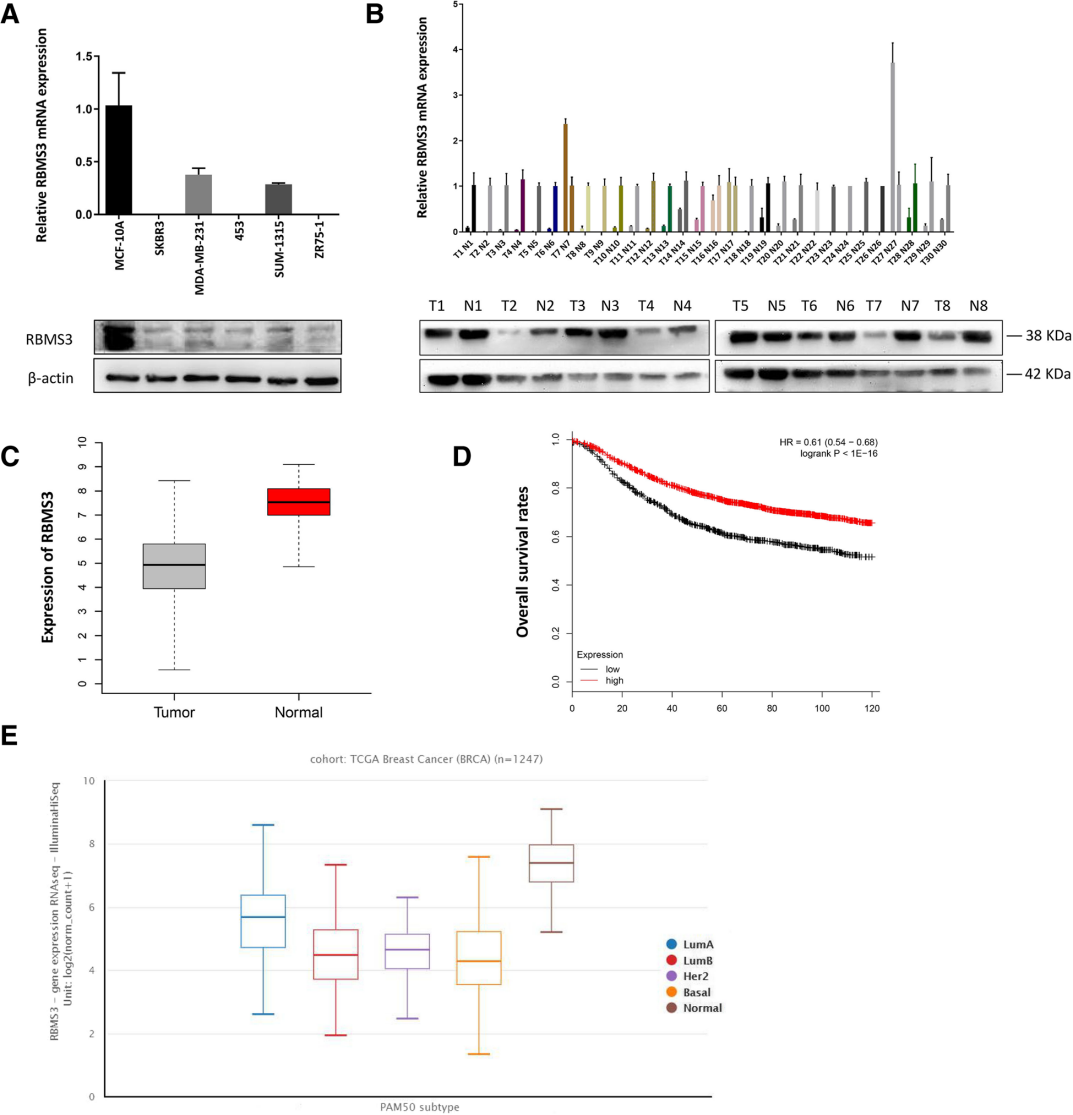
RBMS3 was downregulated in human breast tumors and correlated with poorer prognosis. **a** RBMS3 was downregulated in breast cancer cells. The expression levels of RBMS3 in breast cancer cell lines SKBR3, MDA-MB-231, MDA-MB-453, SUM-1315 and ZR75–1 were detected by Western blot and qRT-PCR, and the non-tumorigenic cell line MCF-10A were used as control. **b** RBMS3 expression was lower in breast cancer tissues. qRT-PCR and western blot were used to detect the expression of RBMS3 in breast cancer tissues and the corresponding adjacent tissues (Reviewer #1 comment 4). The expression of RBMS3 in breast cancer tissues were normalized to the corresponding adjacent tissues. **c** Expression of RBMS3 in the TCGA Breast Cancer (BRCA) database, including 1247 samples, *p*<0.001.(Reviewer #1 comment 2) (**d**) Kaplan-Meier overall survival curve exhibited patients with breast cancer expressing high (red) levels of RBMS3 had better prognosis than those low (black) levels of RBMS3. Including 3955 samples, *P* < 0.05 by log rank test (Reviewer #1 comment 2). The Affymetrix ID of RBMS3 is 206767_at. (Reviewer #2 comment 2) (**e**) RBMS3 was downregulated in the four subtypes of breast cancer compared to normal

Breast cancer cell lines and tissue samples were used to detect the expression level of RBMS3, followed by western blot and qRT-PCR analysis. Figure 1a indicated RBMS3 showed low expression levels in MDA-MB-231, MDA-MB-453, SUM-1315, SKBR3 and ZR-75-1 cell lines, compared to the non-tumorigenic epithelial cell line MCF-10A. Intriguingly, we found that MDA-MB-231 has much higher expression level of RBMS3 than others. We thought that it might be due to other more powerful genes than RBMS3 in MDA-MB-231 cell, which might contributed to the stronger migratory and invasive ability of MDA-MB-231 cell (Reviewer #1 comment 1). Figure 1b showed that RBMS3 was downregulated in breast cancer samples, compared to the paired normal samples, both in mRNA and protein levels (Reviewer #1 comment 4). In addition, the Cancer Genome Atlas (TCGA) data indicated that RBMS3 had lower expression in tumor tissues than in normal tissues (Fig. 1c). Further analyses suggested that the expression of RBMS3 in the normal breast tissues (Reviewer #1 comment 3) were higher than those in luminal A, luminal B, HER2- enriched and Basal-like subtypes (Fig. 1e). Kaplan-Meier analysis revealed that upregulation of RBMS3 was correlated with better prognosis (HR = 0.61) in breast cancer patients (Fig. 1d).

### RBMS3 repressed the migration of breast cancer cells in vitro and in vivo

**Fig. 2:**
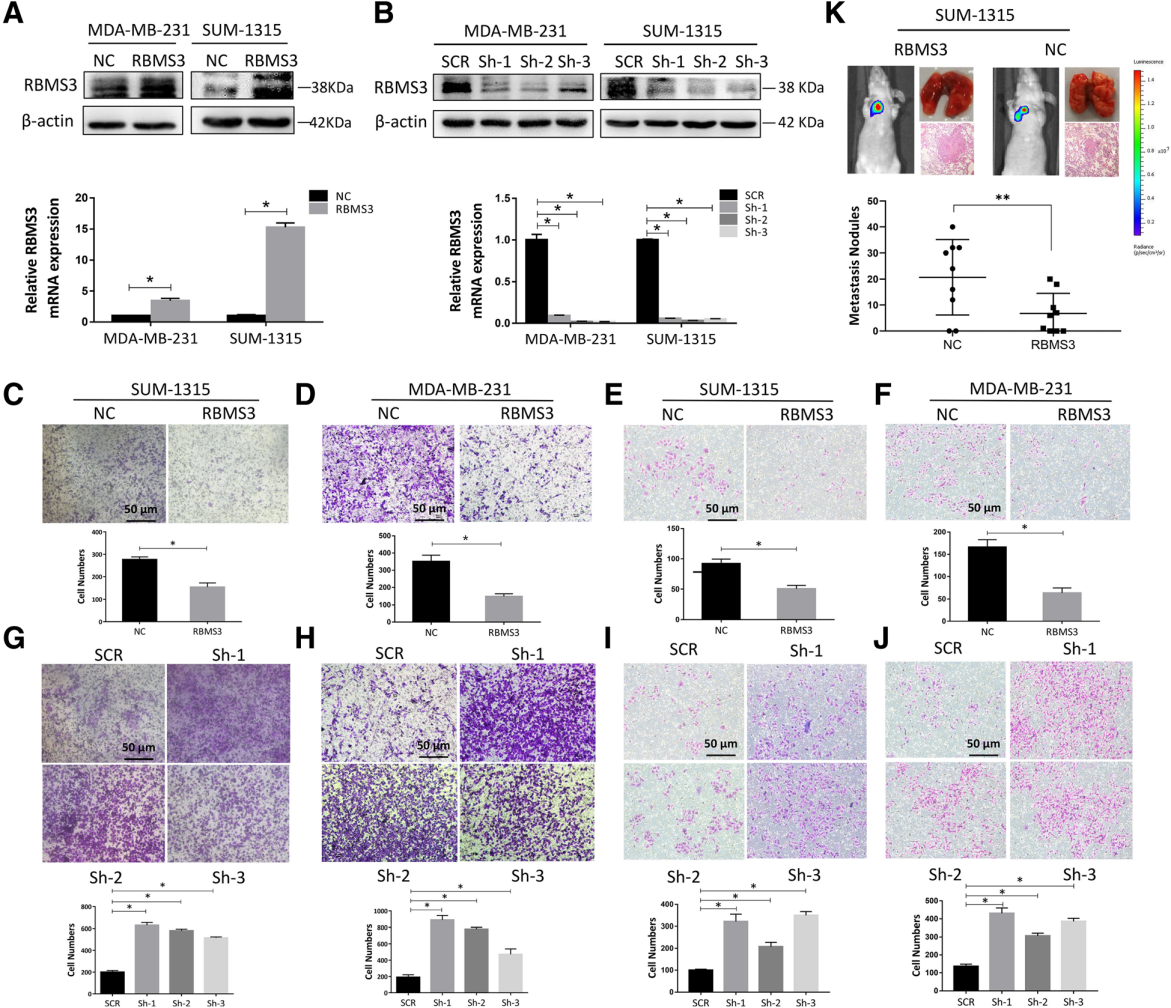
RBMS3 inhibited the migration invasion of breast cancer cells in vitro and in vivo. **a**, **b** SUM-1315 and MDA-MB-231 cell lines were respectively transfected with lentivirus to overexpress (RBMS3) or knocked down (sh-1, sh-2, sh-3) RBMS3 expression. Western blot and qRT-PCR were applied to verify transfection efficiency. **c**-**j** RBMS3 inhibited the invasion and migration of breast cancer cells. **c**, **g**, **e**, **i** Transwell experiment was used to detect the invasion and migration ability of SUM-1315 cells. The lower panel of each picture showed migrating and invading numbers of SUM-1315 cells. Transwell assay performed in MDA-MB-231 cells were analyzed as in Fig. 2d, h, f, and j. Scale bars, 50 μm. (Reviewer #1 comment 5) Data were shown as mean ± SEM, *P < 0.05. **k** RBMS3 inhibited the lung metastasis in breast cancer cells. Representative bioluminescence images of the mice and HE staining of lung section showed the sizes and numbers of lung colonization in the RBMS3-overexpressed group and the control group, respectively. Metastasis nodules plot was generated by the H&E-stained lung sections of nude mice (*n* = 9). Data were shown as mean ± SEM, *P < 0.05, **P<0.001 (Reviewer #1 comment 6). SCR = Scrambled control; Sh = Short hairpin; NC = Negative control

To better understand the effect of RBMS3 on the metastasis of breast cancer, SUM-1315 and MDA-MB-231 cells were transfected with lentivirus to stably overexpress or repress the expression of RBMS3. qRT-PCR and western blot were performed to verify the transfection efficiency (Fig. 2a and b). Then, transwell assays was carried out to examine whether RBMS3 had the ability to suppress breast cancer cell migration. The migrating number of SUM-1315 and MDA-MB-231 cells dropped by 1.5 to 2 fold after the overexpression of RBMS3 (Fig. 2c, d, e and f). In contrast, knockdown of RBMS3 significantly enhanced the cell migration by 2.5 to 4.5 fold (Fig. 2g, h, i and j). All the results indicated that RBMS3 could suppress cell migration and invasion in vitro*.* Furthermore, the luciferase labelled SUM-1315 cells were injected into tail veins of nude mice. Figure 2k implied that the number and volume of metastases in RBMS3 overexpression group (RBMS3) were evidently decreased, compared to the control group (NC) (Reviewer 1, comment 6). These data strongly proved that RBMS3 could inhibit breast cancer metastasis in vitro and in vivo*.*

### Transcriptome analysis for RBMS3

**Fig. 3:**
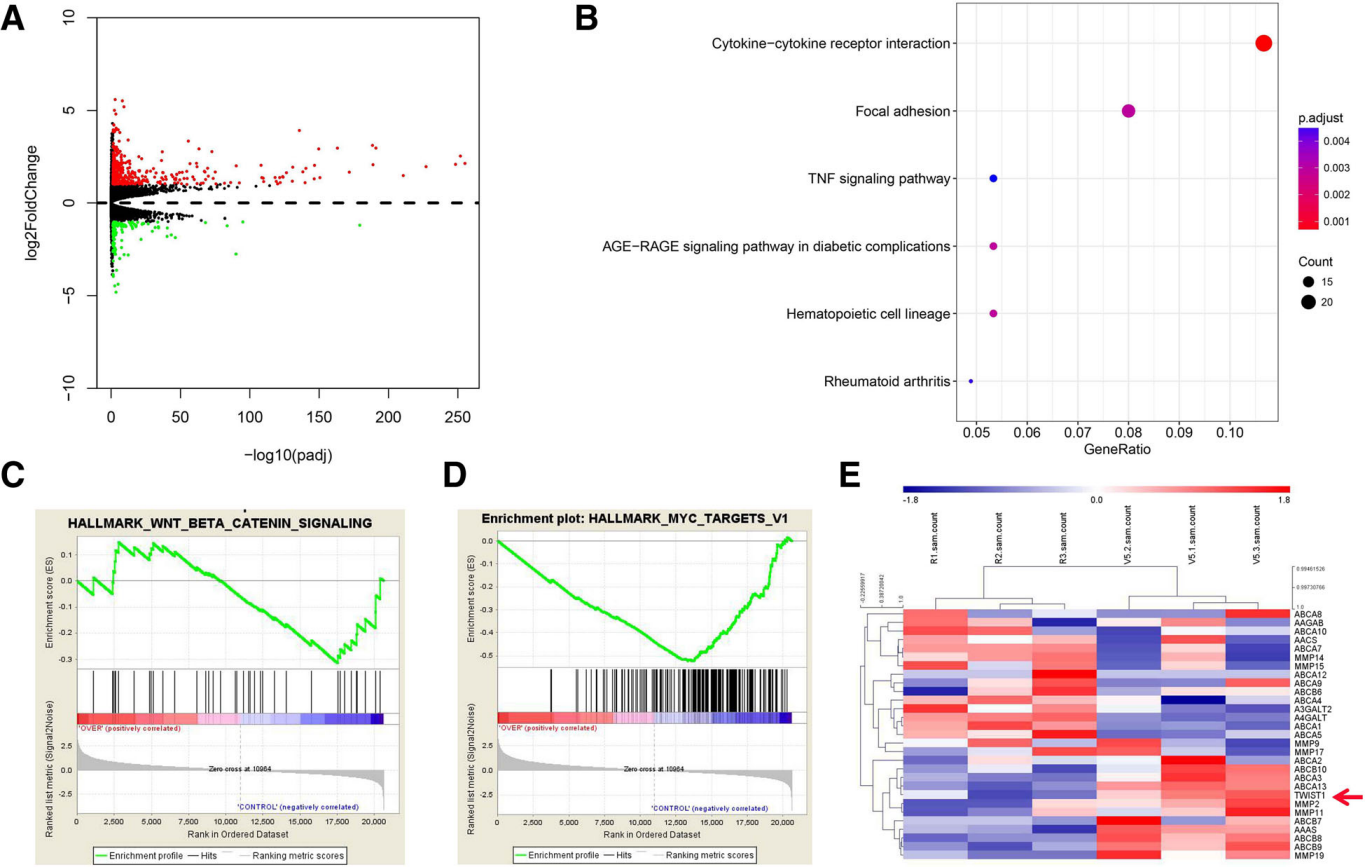
Transcriptome analysis for RBMS3. **a** Volcano plot represented the distribution of mapped transcripts. **b** Enrichment analysis of the KEGG pathway. The size and color of the dots represented the number of enriched genes and the adjusted *P* values, respectively. **c**, **d** Gene Set Enrichment Analysis (GSEA) was used to analyze the distribution of differentially expressed genes in WNT and MYC pathways. **e** Heat map represented the down-regulated and upregulated genes measured in SUM-1315 cells. R1, R2, R3 and V1, V2 and V3 represented RBMS3-overexpressed group and the control group, respectively. The red arrow indicated that Twist1 was downregulated in the RBMS3-overexpressed group

To further investigate the molecular mechanism of RBMS3 involved in the metastasis of breast cancer, the RBMS3 overexpressing group and the control group of SUM-1315 cells were selected for transcriptome sequencing to screen the potential target of metastasis. Figure 3b indicated that RBMS3 was strongly correlated with cytokine receptor interaction, focal adhesion. Furthermore, RBMS3 was found to be involved in the Wnt and Myc signaling pathway by using Gene Set Enrichment Analysis (GSEA) (Fig. 3c and d). As a result, Twist1 was identified as the downstream target of RBMS3 not only because it was one of the most downregulated gene in RBMS3 overexpressed cells, but also known as the metastasis-associated biomarker (Fig. 3e).

### RBMS3 regulated Twist1 expression

**Fig. 4:**
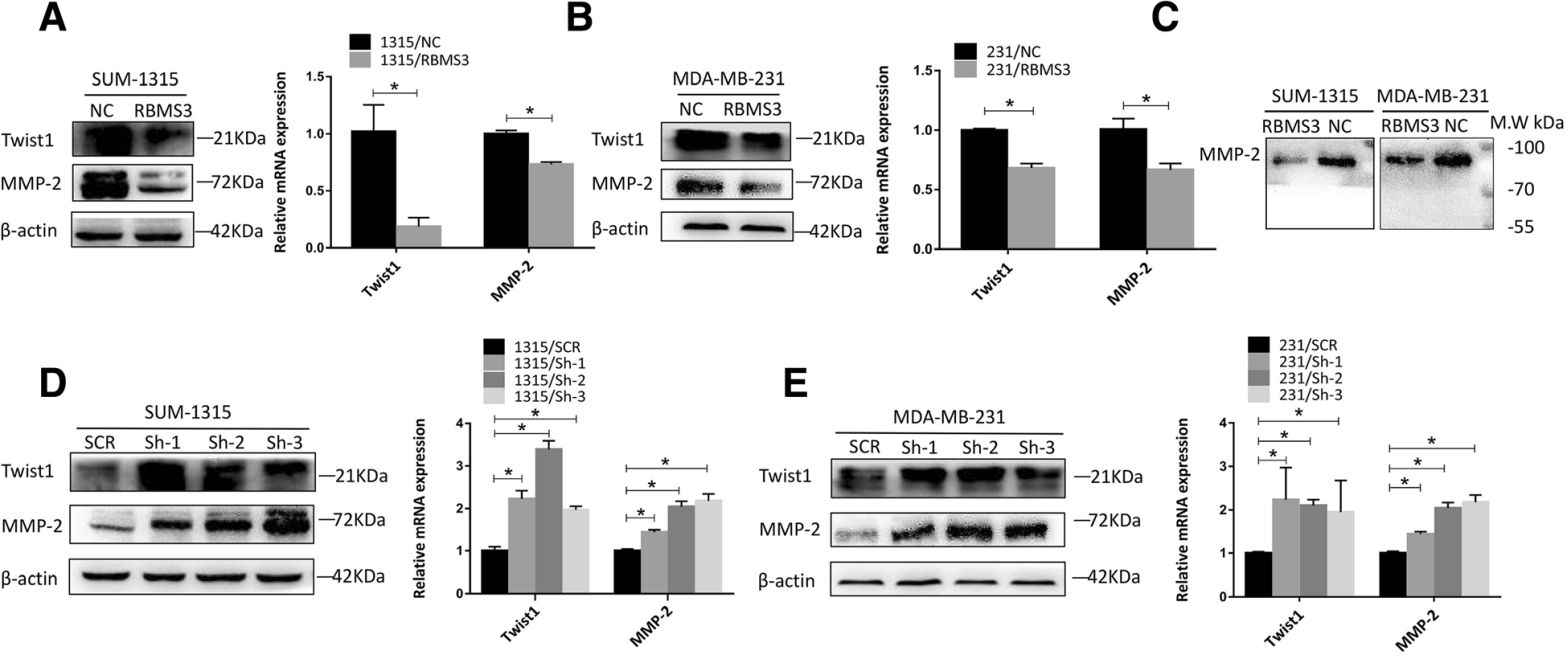
RBMS3 regulated Twist1 and MMP-2 expression. **a**, **b** In SUM-1315 and MDA-MB-231 cell lines, overexpression of RBMS3 inhibited the expression of Twist1, MMP-2. Western blot was used to detect the expression of Twist1, MMP-2 at the protein level. qRT-PCR was applied to examine the expression of Twist1, MMP-2 at the mRNA level. **d**, **e** Knockdown of RBMS3 contributed to the elevated expression of Twist1 and MMP-2. Similar methods were conducted as described in (**a**, **b**). **c** Overexpression of RBMS3 inhibited the expression of secreted MMP-2 protein in SUM-1315 and MDA-MB-231 cell lines. Western blot was used to detect the expression of MMP-2 in conditioned media. Data were shown as mean ± SEM, *P < 0.05. SCR = Scrambled control; Sh = Short hairpin; NC = Negative control

Figure 4a and d showed that ectopic expression of RBMS3 significantly decreased Twist1, MMP-2. While, knockdown of RBMS3 promoted the expression of Twist1 and MMP-2 in SUM-1315 cells. Similar results were observed in MDA-MB-231 cells (Fig. 4b and e). Additionally, to investigate if RBMS3 could reduce the extracellular levels of MMP2, western blot was conducted to examine the media conditioned by RBMS3 group and NC group of SUM-1315 and MDA-MB-231 cells. Figure 4c suggested that extracellular MMP2 levels were decreased in RBMS3 group.

### RBMS3 destabilized Twist1 transcript by binding to its mRNA

**Fig. 5:**
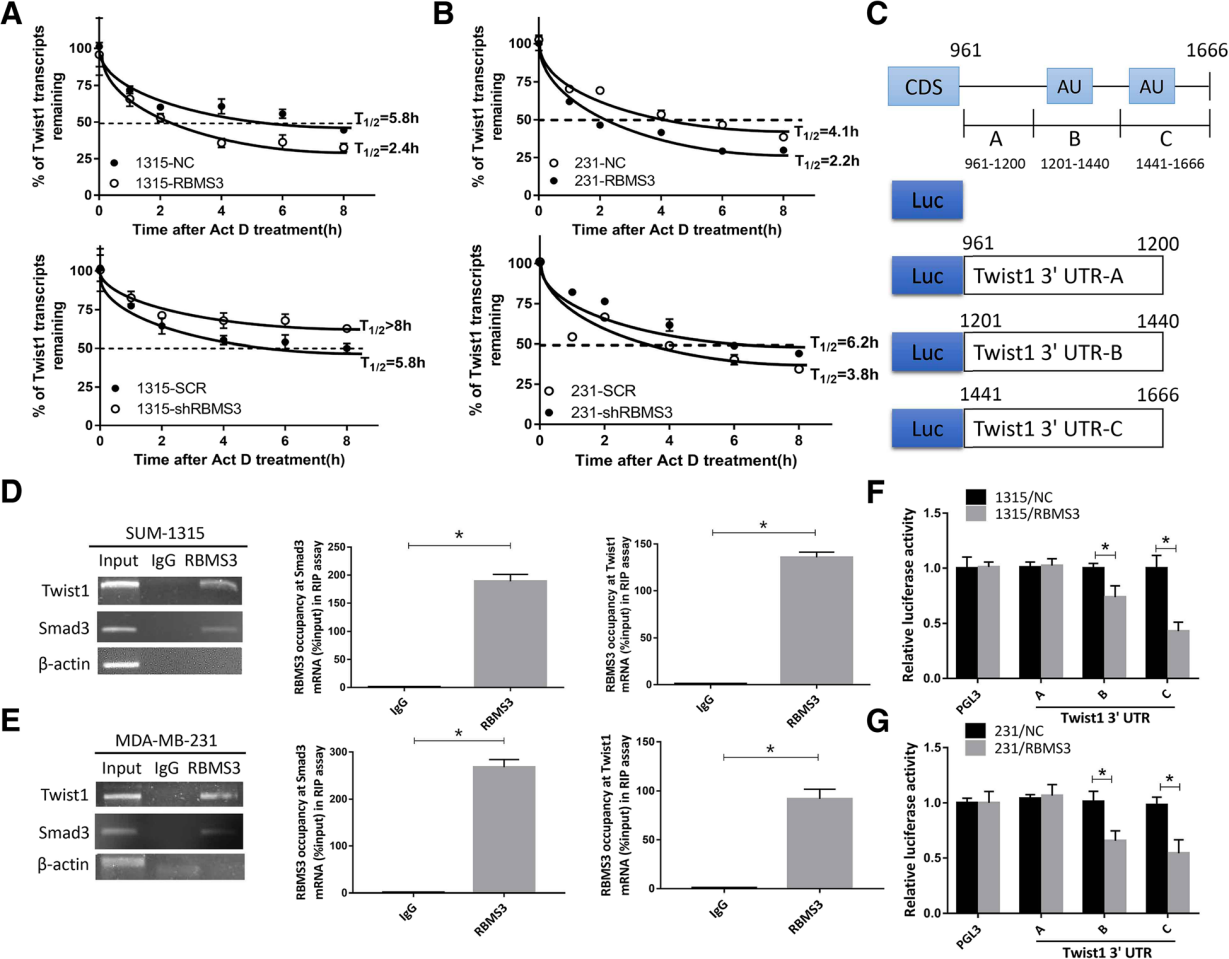
RBMS3 destabilized Twist1 transcript by directly binding to the 3′-UTR of Twist1 mRNA. **a**, **b** in SUM-1315 and MDA-MB-231 cell lines, RBMS3 overexpression shortened the half-life of Twist1 mRNA, while knockdown of RBMS3 prolonged the halflife of Twist1 mRNA. Overexpression (RBMS3) and control cells (NC), knockdown (shRBMS3) and the control (SCR) were treated with Act D at a concentration of 5 μg/ml. The total RNA were extracted at 0, 1, 2, 4, 6, and 8 h, respectively, and then followed by qRT-PCR analysis. **d**, **e** SUM-1315 and MDA-MB-231 cells lysates were immunoprecipitated with RBMS3 or IgG antibody and analyzed by using RT-PCR and qRT-PCR to detect Twist1 and Smad3 transcript levels. **c** Schematic diagram of various regions in the 3′-UTR of Twist1 mRNA. **f**, **g** The reporter containing Twist1 3′-UTR-B, −C was decreased by overexpression of RBMS3 in SUM-1315 and MDA-MB-231 cells. Data were shown as mean ± SEM, *P < 0.05

RBMS3 overexpressing and the control cells were treated with actinomycin-D (Act D, 5 mg/ml) for differential times. Figure 5a indicated that ectopic expression of RBMS3 decreased the half-life of Twist1 mRNA from 5.8 to 2.4 h. Knockdown of RBMS3 increased the half-life of Twsit1 t mRNA from 5.8 to > 8 h in SUM-1315 cells. Similar results were confirmed in MDA-MB-231 cells (Fig. 5b). These results suggested that RBMS3 could decrease Twist1 expression via regulating its mRNA stability.

### RBMS3 directly bound to the 3′-UTR of Twist1 mRNA

Furthermore, we examined whether RBMS3 could bind to Twist1 mRNA directly. RNA immunoprecipitation assay was carried out, followed by RT-PCR and qRT-PCR in SUM-1315 and MDA-MB-231 cells. The results showed that Twist1 was detected in RBMS3 and Input, whereas not in IgG. Smad3 was presented as a positive control according to the previous study [28], and β-actin was detected as a negative control as it was not capable of binding to RBMS3 (Fig. 5c and d). It suggested that RBMS3 could bind physically to Twist1 mRNA. To further explore if RBMS3 could specifically binds to AU-rich elements (AREs) in the 3′-UTR of Twist1 mRNA, luciferase reporter assay was carried out with pGL3 reporter containing A, B, C, and D regions of the 3′-UTR. As the schematic diagram suggested, 3′-UTR-B and C contained AREs while 3′-UTR-A did not. The histograms indicated that the reporter carrying 3′-UTR-C and B exhibited stronger luciferase activity in RBMS3 overexpressing of SUM-1315 and MAD-MB-231 cells than that of 3′-UTR-A. These results proved that RBMS3 could directly bind to AREs in the 3′-UTR of Twist1 mRNA, thereby inhibiting its expression.

### Rbms3 inhibited Twist1-induced migration and metastasis in vitro and in vivo

**Fig. 6:**
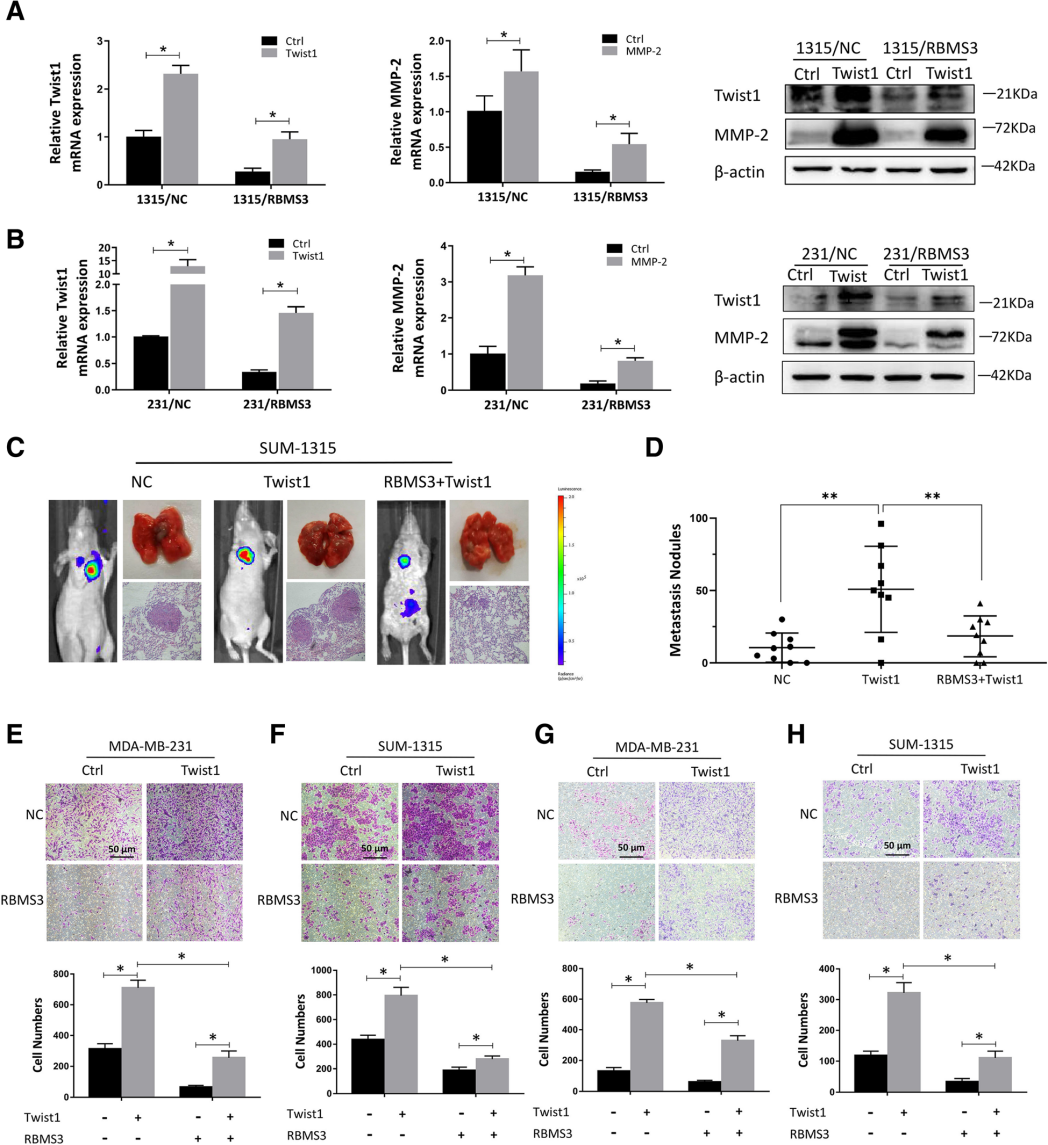
RBMS3 inhibited Twist1-induced migration and metastasis in vitro and in vivo. **a**, **b** RBMS3-overexpressed and the control groups of SUM-1315 and MDA-MB-231 cells were transfected to overexpress Twist1, followed by western blots and qRT-PCR examination. **c**-**h** RBMS3 reversed the invasion and metastasis of Twist1 induced enhanced breast cancer cells in vitro and in vivo. (E-H) In SUM-1315 and MDA-MD-231 cells, transwell assay was used to detect invasion and migration ability. The lower panel of each picture showed the migrating and invading cell numbers. **c**, **d** Representative bioluminescence images showed the sizes and numbers of lung metastasis. The color scale indicated bioluminescence. Metastasis nodules plot was generated by the H&E-stained lung sections of nude mice (*n* = 9). Data were shown as mean ± SEM, *P < 0.05, **P<0.001 (Reviewer #1 comment 6)

Twist1 is known as a major transcription factor which can promote cell motility, migration, and invasion in breast cancer cells. To investigate the effect of RBMS3 on Twist1-induced cell migration and invasion, RBMS3 overexpressing and the control groups of SUM-1315 and MDA-MB-231 cells were transfected to overexpress Twist1, followed by western blot and qRT-PCR examination (Fig. 6a and b). Figure 6e and g showed that Twist1-overexpressed group exhibited a stronger ability of migration and invasion in MDA-MB-231 cells, while cell migration and invasion was significantly inhibited by the overexpression of RBMS3. Similar results were obtained in SUM-1315 cells (Fig. 6f and h). Furthermore, 1315-Twist1, 1315-Twist1-ctrl, and 1315-Twist1 + RBMS3 were injected into tail veins of nude mice. Figure 6c and d indicated that ectopic expression of Twist1 evidently increased lung metastases formed in size and number, while overexpression of RBMS3 strongly reversed the formation of metastases. All the results demonstrated that RBMS3 could inhibit Twist1-induced migration and metastasis in vitro and in vivo*.*

## Discussion

In the present study, we revealed that RBMS3 could inhibit breast cancer metastasis in vitro and in vivo. Using transcriptome sequencing analysis to screen the differential expression genes affected by RBMS3, we found that RBMS3 could regulate Twsit1 expression via stabilizing its mRNA by directly binding to the 3′-UTR of Twist1 mRNA. Furthermore, suppression of Twist1 contributed to the downregulation of MMP2, which was able to degrade and remodel ECM.

RBMS3 belongs to c-myc single-strand binding protein family, which includes three members, RBMS1, RBMS2, and RBMS3 [17]. It has been a long time that RBMS3 was discovered, while the study on its biological function and underlying mechanism are far from elucidated. RBMS3 was found to suppress tumor angiogenesis by regulating HIF1α expression [29], which implicated the strong correlation between RBMS3 and tumor metastasis. The present study gave strong evidences to show that RBMS3 could inhibit breast cancer metastasis. Knockdown of RBMS3 increased cancer cells migration ability, while overexpression of RBMS3 repressed cancer cell migration and invasion in vitro and inhibited lung metastases in vivo. Moreover, upregulation of RBMS3 was correlated with better prognosis in breast cancer patients.

Transcriptome analysis revealed that RBMS3 was associated with focal adhesion. Twist1 was then identified as the downstream target of RBMS3 related to its metastasis inhibiting ability. Moreover, overexpression of RBMS3 decreased Twist1 expression, while RBMS3 knockdown increased Twist1 expression. RBMS3 was capable of decreasing Twist1 mRNA stability by shorten its half-life. Furthermore, we verified that RBMS3 could directly bind to Twist1 mRNA by RIP assay. Previous study indicated that RBMS3 could regulate target gene expression by strongly binding to AREs in the 3′-UTR of target gene mRNAs [30]. Consistent with this, we also found that RBMS3 could directly bind to fragment B and C in 3′-UTR of Twist1 mRNA, which contributed to the destability of Twist1 mRNA. Hence, we revealed a novel mechanism that RBMS3 could posttranscriptionally regulate Twsit1 expression in breast cancer.

During cancer metastasis, the initial and the most critical process is detachment of tumor cells from the primary site and invasion into adjacent tissue. Accordingly, ECM remodeling, which could promote cell differentiation, migration, and invasion by regulating matrix deposition and matrix stiffness [31, 32], is involved in and necessary for this process. Twist1 could induce ECM remodeling by activating cancer-associated fibroblast to synthesize and secret high levels of ECM proteins [33, 34], such as MMP2, which was proved to be related to tumor formation, metastasis, and responsible for high mortality breast and poor prognosis in breast cancer patients [35, 36]. Here, we found that ectopic expression of Twist1 could induce MMP2 expression and promote breast cancer migration, invasion and lung metastasis. Whereas, upregulation of RBMS3 could alleviate Twist1-induced MMP2 expression and abrogate migration, metastasis ability of breast cancer cells, correspondingly. Our study implied that RBMS3-mediated decrease in Twist1 expression played a crucial role in the breast cancer metastasis process.

## Conclusions

In summary, we demonstrated that the RBMS3 was a novel target for metastasis inhibition in breast cancer. We also provided a novel mechanism of the RBMS3/Twsit1/MMP2 axis in the regulation of breast cancer invasion and metastasis, which may become a potential molecular marker for breast cancer treatment.

## Abbreviations

3′-UTR: 3′-untranslated region
Angptl4: Angiopoietin-like-4
ECM: Extracellular matrix
EGFR: Epidermal growth factor receptor
EMT: Epithelial mesenchymal transition
IL-6: Interleukin 6
MMP2: Matrix metalloproteinase 2
RBMS3: RNA binding motif single stranded interacting protein 3
RBPs: RNA binding proteins
TGF-β: Transforming growth factor-β
