# Correction to: The RNA binding protein RBMS3 inhibits the metastasis of breast cancer by regulating Twist1 expression

**DOI:** 10.1186/s13046-019-1509-0

**Published:** 2020-01-27

**Authors:** Lei Zhu, Pei-Wen Xi, Xiao-Xia Li, Xi Sun, Wen-Bin Zhou, Tian-Song Xia, Liang Shi, Yue Hu, Qiang Ding, Ji-Fu Wei

**Affiliations:** 1 Jiangsu Breast Disease Center, the First Affiliated Hospital with Nanjing Medical University, 300 Guangzhou Road, Nanjing, 210029 China; 2 Research Division of Clinical Pharmacology, the First Affiliated Hospital with Nanjing Medical University, 300 Guangzhou Road, Nanjing, 210029 China; 3 Department of Critical Care Medicine, The First Affiliated Hospital with Nanjing Medical University, 300 Guangzhou Road, Nanjing, 210029 China

**Correction to: J Exp Clin Cancer Res (2019) 38:105**

**https://doi.org/10.1186/s13046-019-1111-5**

In the original publication of this article [1], the molecular weight of RBMS3 was incorrectly noted as 38 KDa within Fig, 1a, Fig. 2a and Fig. 2b. The figures have been updated to list the correct molecular weight of RBMS3 as 41 KDa.

**Fig. 1:**
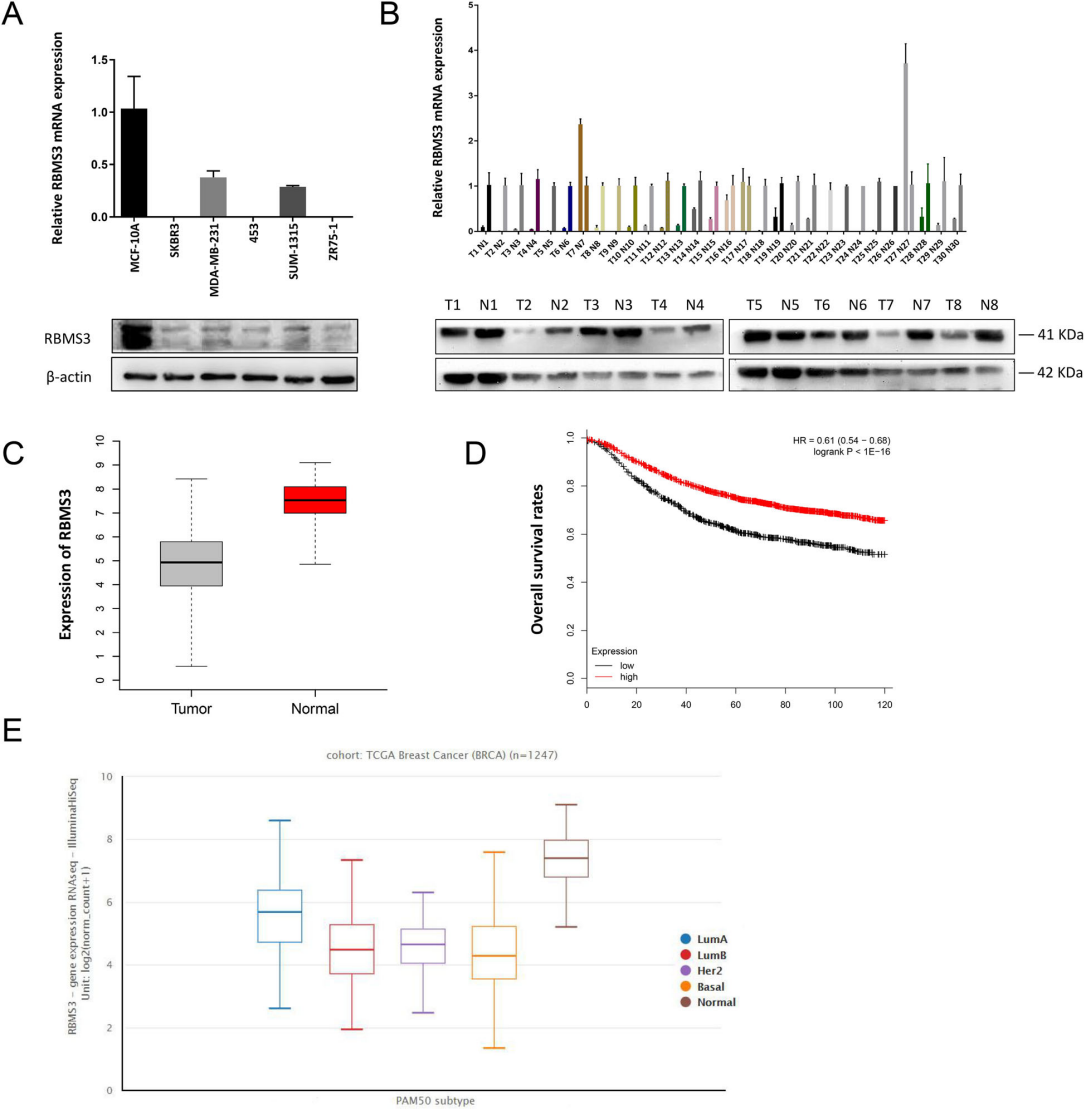
RBMS3 was downregulated in human breast tumors and correlated with poorer prognosis. **a** RBMS3 was downregulated in breast cancer cells. The expression levels of RBMS3 in breast cancer cell lines SKBR3, MDA-MB-231, MDA-MB-453, SUM-1315 and ZR75–1 were detected by Western blot and qRT-PCR, and the non-tumorigenic cell line MCF-10A were used as control. **b** RBMS3 expression was lower in breast cancer tissues. qRT-PCR and western blot were used to detect the expression of RBMS3 in breast cancer tissues and the corresponding adjacent tissues (Reviewer #1 comment 4). The expression of RBMS3 in breast cancer tissues were normalized to the corresponding adjacent tissues. **c** Expression of RBMS3 in the TCGA Breast Cancer (BRCA) database, including 1247 samples, *p*<0.001.(Reviewer #1 comment 2) (**d**) Kaplan-Meier overall survival curve exhibited patients with breast cancer expressing high (red) levels of RBMS3 had better prognosis than those low (black) levels of RBMS3. Including 3955 samples, *P* < 0.05 by log rank test (Reviewer #1 comment 2). The Affymetrix ID of RBMS3 is 206767_at. (Reviewer #2 comment 2) (**e**) RBMS3 was downregulated in the four subtypes of breast cancer compared to normal

**Fig. 2:**
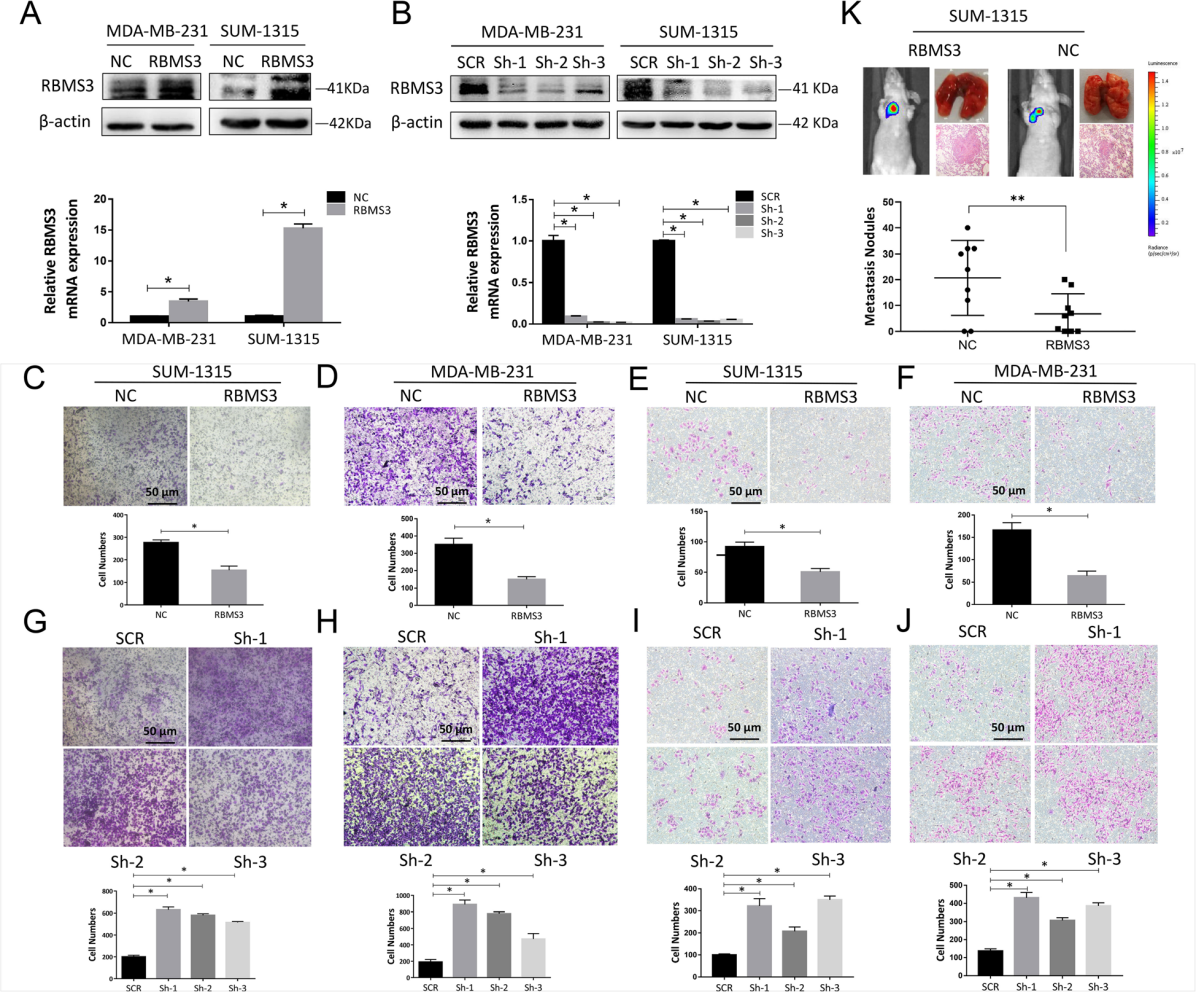
RBMS3 inhibited the migration invasion of breast cancer cells in vitro and in vivo. **a**, **b** SUM-1315 and MDA-MB-231 cell lines were respectively transfected with lentivirus to overexpress (RBMS3) or knocked down (sh-1, sh-2, sh-3) RBMS3 expression. Western blot and qRT-PCR were applied to verify transfection efficiency. **c**-**j** RBMS3 inhibited the invasion and migration of breast cancer cells. **c**, **g**, **e**, **i** Transwell experiment was used to detect the invasion and migration ability of SUM-1315 cells. The lower panel of each picture showed migrating and invading numbers of SUM-1315 cells. Transwell assay performed in MDA-MB-231 cells were analyzed as in Fig. 2d, h, f, and j. Scale bars, 50 μm. (Reviewer #1 comment 5) Data were shown as mean ± SEM, *P < 0.05. **k** RBMS3 inhibited the lung metastasis in breast cancer cells. Representative bioluminescence images of the mice and HE staining of lung section showed the sizes and numbers of lung colonization in the RBMS3-overexpressed group and the control group, respectively. Metastasis nodules plot was generated by the H&E-stained lung sections of nude mice (*n* = 9). Data were shown as mean ± SEM, *P < 0.05, **P<0.001 (Reviewer #1 comment 6). SCR = Scrambled control; Sh = Short hairpin; NC = Negative control

The authors sincerely apologize for the inconvenience caused to the readers.
